# Paranormal belief and conspiracy theory endorsement: variations in adaptive function and positive wellbeing

**DOI:** 10.3389/fpsyg.2025.1519223

**Published:** 2025-02-27

**Authors:** Neil Dagnall, Andrew Denovan, Kenneth Graham Drinkwater, Álex Escolà-Gascón

**Affiliations:** ^1^School of Psychology, Manchester Metropolitan University, Manchester, United Kingdom; ^2^School of Psychology, Liverpool John Moores University, Liverpool, United Kingdom; ^3^Department of Quantitative Methods and Statistics, Comillas Pontifical University, Madrid, Spain

**Keywords:** paranormal belief, conspiracy theory endorsement, positive wellbeing, active and avoidant coping, meaning in life

## Abstract

Recent studies report that paranormal belief and conspiracy theory endorsement are differentially related to factors allied to positive wellbeing (e.g., meaning in life and coping behaviours). Since these findings derive from correlational studies using cross sectional designs, researchers need to undertake further investigation to establish outcome robustness. Accordingly, the present study used a multiple time point design. Respondents (*N* = 1,158) completed measures on three occasions, three months apart. While a strong positive association was found between paranormal belief and conspiracist theory endorsement, path analysis revealed divergent relationships with positive wellbeing outcomes. Specifically, paranormal belief predicted greater levels of positive wellbeing over time (meaning in life and social identity), whereas conspiracy theory endorsement predicted only social identity. Consideration of mediation effects revealed that paranormal belief prognosticated greater presence of meaning in life via links with active coping and positive outlook. Additionally, avoidant coping positively mediated the paranormal belief-search for meaning in life relationship. Conspiracy theory endorsement predicted greater social identity via avoidant coping. Findings indicated that paranormal belief and conspiracy theory endorsement were differentially related to positive wellbeing outcomes. Regarding paranormal belief, the construct was concomitantly attendant with passive and active psychological functions. The association with avoidant coping suggested that in some circumstances supernatural credence enables believers to avert attention from problems.

## Introduction

Despite lacking scientific verification, paranormal belief and conspiracy theory endorsement persist within contemporary Western society (Georgiou et al., 2021; Williams et al., 2022). Scholarly interest in these unorthodox convictions arose because of their prevalence and capacity to adversely influence personal and social attitudes/behaviours. Illustratively, paranormal credence can inappropriately inform believers’ health choices (Denovan et al., 2024a; Denovan et al., 2024b) and conspiracy theories routinely undermine modern medicine (i.e., vaccine uptake) (Jolley et al., 2020). In instances such as these, ideations are problematic and highly resistant to change (i.e., unfalsifiable and unaffected by refutation).

Though researchers have extensively investigated paranormal belief and conspiracy theory endorsement, few studies compare their adaptive functions. While adaptation generally refers to the process by which individuals maintain wellbeing and functionality by adjusting to changing environmental demands (Lazarus and Folkman, 1984), this study used the term to denote positive wellbeing. Due to an absence of studies examining relationships between paranormal belief, conspiracy theory endorsement and positive wellbeing, this theoretical domain remains under researched and poorly comprehended. Thus, while the extant literature provides insights, lack of conceptual coherence coupled with methodological limitations (i.e., variations in construct operationalisation and use of different measurement instruments) undermine findings (e.g., Lobato et al., 2014; Dyrendal et al., 2021; van Prooijen et al., 2022). Moreover, researchers have focused on paranormal belief and conspiracy theory endorsement commonality to the neglect of differences (e.g., Lobato et al., 2014; van Prooijen et al., 2022). Accordingly, this paper extends inquiry by examining how paranormal belief and conspiracy theory endorsement relate differentially to positive wellbeing.

To achieve this, it is necessary to consider both constructs. Paranormal belief designates validation of supernatural propositions (e.g., powers, forces, and entities) generated by non-scientific communities that people capable of rational thought and reality testing ratify, despite lack of scientific authentication (see Irwin, 2009). While this is the prevailing academic perspective it is important to acknowledge that scholars in other domains (i.e., parapsychology) view the scientific perception of consciousness as reductionist (Kelly and Kelly, 2007; Kelly et al., 2015; Wahbeh et al., 2022).

The advantage of the psychological explication used in the present study, is that it recognizes the prosaic nature of belief, which is widespread in non-clinical populations (Dagnall et al., 2016). Specifically, it acknowledges that paranormal belief is a naturally occurring feature of human cognition arising from flawed/biased information processing (e.g., faulty thinking and overreliance on subjective data) (Irwin et al., 2012a; Irwin et al., 2012b).

Reality testing deficits refer to the inability to adequately appraise belief legitimacy. Reality testing deficits occur when individuals fail to distinguish between external and internal generated sources of information. Consequently, their judgments draw heavily on subjective, intuitive evidence (i.e., personal insights, feelings). A manifestation of this is ontological confusion (Rizeq et al., 2021), where individuals fail to correctly discriminate between superordinate categories (i.e., mental and physical) (Dyrendal et al., 2021).

Since investigators define conspiracy in myriad ways, the construct is best delineated using prevailing canonical themes (e.g., premeditation, deception, intention, and manipulation). Collectively, these coalesce to form narratives where the purposeful exploitation of power adversely impacts individuals and society (Drinkwater et al., 2023).

The notion that paranormal belief and conspiracy theories perform similar psychological functions arises from consistent reporting of a moderate/high positive correlation between the two constructs (e.g., Darwin et al., 2011; Dyrendal et al., 2021). This supposition is strengthened by similar relationships with allied variables (e.g., schizotypy; Dagnall et al., 2024a), and the presence of common manifest features (i.e., intuitive thinking, reality testing deficits, and the tendency to form conclusions that exceed the sum of knowledge/evidence strengthen this view) (Lobato et al., 2014).

Noting construct overlap, some theorists have located paranormal belief and conspiracy theory endorsement, alongside pseudoscience, within a generic category. Notable instances are scientifically unsubstantiated (Mill et al., 1994) and epistemically unwarranted beliefs (Lobato et al., 2014). While these classifications offer theoretical parsimony and denote the generalized tendency to adopt self-generated, internally verified worldviews (Jastrzębski and Chuderski, 2022), they are reductionist to the extent that they neglect differences potentially related to adaptive functioning.

To expound these, Dagnall et al. (2024b) used the taxonomy of rational thinking problems proposed by Stanovich et al. (2008). Within this, paranormal belief aligns with defective understanding of scientific knowledge, whereas conspiracy endorsement corresponds to contaminated mindware. In the latter case, problematic data disenables critical appraisal, encouraging egocentric thought, and promoting acceptance of maladaptive conditioned beliefs (Bensley et al., 2020). Consistent with this distinction, paranormal belief and conspiracy theory endorsement demonstrate divergent relationships with perceptions of the world. Paranormal credence associates positively with belief in a just world (i.e., the conviction that life is fair) and negatively with competitive worldview (i.e., winning is everything and interactions represent struggles over power and resources).

In comparison, conspiracist beliefs correlate positively with competitive worldview, dangerous worldview (i.e., regard existence as threatening and perilous), and zero-sum game belief (i.e., observe resources as finite and think gains result from the exploitation of others) (Grigoryev and Gallyamova, 2023). These variances suggest that conspiracy theory endorsement (relative to paranormal belief) reflects a negative perception of the world. This conclusion aligns with the finding that paranoid ideation is strongly affiliated with conspiracy endorsement, whilst paranormal belief is not (Darwin et al., 2011).

Acknowledging these dissimilarities, Dagnall et al. (2024b) used network analysis to examine relationships between paranormal belief, conspiracy theory endorsement, and positive wellbeing. The observed pattern of connections indicated that paranormal belief, via its connection with self-esteem, linked positively to affirmative wellbeing (presence of meaning in life, active coping, and satisfaction with life) and negatively to factors related to reduced wellbeing (avoidant coping and search for meaning in life). Conspiracy theory endorsement did not connect with positive wellbeing outcomes. Collectively, the network signified that beliefs affiliated with lower self-esteem were likely to reflect poorer/reduced psychological health. Based on the emergent network, Dagnall et al. (2024b) concluded that while endorsement of paranormal belief and conspiracy theories overlapped, they perform distinct psychological functions.

Given the exploratory nature of the Dagnall et al. (2024b) study and the fact that theorists have concerns about the replicability of networks (Hevey, 2018), this paper further assessed adaptive differences between paranormal belief and conspiracy theory endorsement. This was necessary to assess the stability, robustness, and generalizability of the network model. Accordingly, this study more directly evaluated the link between paranormal belief, coping, and positive wellbeing. Explicitly, tested the finding that paranormal belief influences factors associated with positive wellbeing (e.g., meaning in life) via its link with forms of coping (active and avoidant). This was necessary due to the cross-sectional nature of the Dagnall et al. (2024b) study, which prevented consideration of the interaction (influence) between paranormal belief and coping.

Addressing this limitation the current study used a multiple time point approach since it would enable an explicit assessment of variable order within the paranormal belief-wellbeing relationship. Accordingly, the model tested in this study evaluated relationships between paranormal belief, conspiracy theory endorsement, mediators of outlook (i.e., optimism and pessimism) and coping (active and avoidant), meaning in life (search and presence), and social identity. The inclusion of social identity and optimism was informed by previous research, which indicates that stronger group identification promotes positive expectations for the future, reinforcing psychological resilience and overall wellbeing (Jetten et al., 2017).

Noting that theorists define paranormal belief and conspiracy theory endorsement in differing ways, the present paper assessed these constructs using the most frequently used measures, the Revised Paranormal Belief Scale (RPBS, Tobacyk, 2004) and the Generic Conspiracist Beliefs Scale, GCBS (Brotherton et al., 2013). The RPBS definition is commensurate with the classification used in the present study. Specifically, it classifies paranormality in terms of phenomena inexplicability, as defined by current academic wisdom (i.e., basic limiting principles of science, Broad, 1953; and incompatibility with conventional notions of reality, see Alcock, 1981; Braude, 1978).

Similarly with conspiracy theory endorsement, the GCBS aligns with the thematic perspective adopted in this paper. Particularly, the GCBS focuses on endorsement of abstract conspiratorial notions (i.e., ideations) rather than validation of specific theories. In addition to conceptual alignment, advantages of using the RPBS and GCBS are that they feature prominently in published research, sample a breadth of construct content, and possess established psychometric properties (RPBS, Drinkwater et al., 2017; Dagnall et al., 2023a; GCBS, Drinkwater et al., 2020; Fasce et al., 2022; Dagnall et al., 2023b; Dinić et al., 2024).

## Method

### Sample

The sample comprised 1,158 participants (*M*age = 48.00, range = 18 to 82); 630 males (Mage = 49.26, range = 18 to 74); 521 females (Mage = 47.52, range = 18 to 82); two trans (Mage = 41.00, range = 33 to 49) and five non-binary (Mage = 33.00, range = 18 to 54). In addition to issues of power (minimizing Type II errors), estimates prove unstable if analyses use small samples. Moreover, complicated models require large samples for stable estimates. Hence, Kline (2015) advise a 20:1 ratio of cases to each estimated model parameter.

Relative to the present multiple time point design, a further advantage of a large sample is that it counters attrition, and in doing so ensures the statistical model possesses sufficient degrees of freedom and statistical power. A simulation analysis of the model indicated that 37 parameters existed, suggesting a minimum sample of 740. However, the dropout rate for multiple time point data collation (using panel recruitment) can be as high as 30% (Gustavson et al., 2012). The study sample slightly exceeded the required quantity with the 30% potential drop-out factored in.

Participants completed study measures on three occasions, three months apart. The researchers recruited through Bilendi, an established respondent provider. Bilendi supply quality data that has featured in peer reviewed publications and reports (e.g., Dagnall et al., 2022a; Dagnall et al., 2022b; Judah et al., 2022; Hässig-Wegmann et al., 2024) and is equivalent/superior to traditional face-to-face methods. The researchers requested a gender balanced sample encompassing a broad age range starting at 18 years.

### Measures

The study assessed constructs using self-report measures. Recruitment occurred at three time points. Participants at time point 1 (baseline) completed questionnaires capturing paranormal and conspiracy belief. These represented exogenous variables in the study model.

### Revised Paranormal Belief Scale (RPBS)

The RPBS (Tobacyk, 2004) comprises 26 items, which measure validation of supernatural phenomena. Participants respond to items (e.g., ‘There is a devil’) using a seven-point Likert scale, with options ranging from 1 (strongly disagree) to 7 (strongly agree). The researchers selected the RPBS because it is an established instrument that features prominently in psychological research. Moreover, in comparison with the alternative prevailing measure, the Australian Sheep Goat Scale (ASGS, Thalbourne and Delin, 1993), which assesses only core parapsychological phenomena (i.e., extrasensory perception, psychokinesis, and life after death) (Drinkwater et al., 2018), the RPBS considers a breadth of construct content (i.e., traditional religious belief, psi, witchcraft, superstition, spiritualism, extraordinary life forms, and precognition). Consistent with Irwin (2009), prior to analysis the researchers recoded scores (0–6). Hence, total scores ranged from 0 to 156 with higher scores being indicative of stronger levels of paranormal belief.

### Generic Conspiracist Beliefs Scale (GCB-5)

The GCB-5 (Kay and Slovic, 2023) is a brief measure of conspiratorial ideation, which comprises the highest loading items from each of the five GCBS dimensions (i.e., Government Malfeasance: assertions of criminal conspiracy within government, Extraterrestrial Cover-Up: deception about extraterrestrial existence, Malevolent Global Conspiracies: allegations that secret groups control global events, Personal Well-Being: concerns over health and liberty, and Control of Information: manipulation of information by institutions) (Brotherton and French, 2014). Participants read the presented statements (e.g., ‘New and advanced technology which would harm current industry is being suppressed’) and respond via a 5-point Likert-type scale (1 = definitely not true to 5 = definitely true). Higher scores indicate greater conspiratorial advocacy.

Participants at time point 2 completed scales indexing outlook (i.e., optimism and pessimism) and coping (active and avoidant) three months after completing the baseline measures. The variables captured at time point 2 represented mediators in the statistical model.

### Optimism–Pessimism Short Scale–2 (SOP2)

The SOP2 (Nießen et al., 2022) assesses psychological inclination to optimism via responses to two items (‘Optimists are people who look to the future with confidence and who mostly expect good things to happen. How optimistic are you in general?’ and ‘Pessimists are people who are full of doubt when they look to the future and who mostly expect bad things to happen. How pessimistic are you in general?’, reverse scored), using a 7-point Likert scale (1 = not at all to 7 = very). In the present study rather than using a summated total to indicate overall level of optimism the researchers used item scores as indices of optimism and pessimism. In this context, the pessimism item was not reversed. Higher scores designated greater levels of each construct.

### Brief-COPE

The Brief-COPE (Carver, 1997) measures individual attempts to minimize distress arising from negative life experiences. Within this study, the researchers evaluated active (12 items: e.g., ‘I concentrate my efforts on doing something about it’) and avoidant (12 items: e.g., ‘I refuse to believe that it has happened’) coping. Participants respond to statements using a 4-point Likert type scale (1 = I usually do not do this to 4 = I usually do this a lot). Higher subscale scores designate greater levels of active and avoidant coping.

Three months after time point 2, participants completed scales capturing meaning in life (search and presence), and social identity. These represented outcome (endogenous) variables in the statistical model.

### Meaning in Life Questionnaire (MLQ)

The MLQ (Steger et al., 2006) evaluates perceived sense of purpose via two subscales: presence (5 items, ‘I have discovered a satisfying life purpose’) and search for meaning in life (5 items, ‘I am seeking a purpose or mission for my life’). Presence signifies the perception that life is meaningful, and search denotes an attempt to find or deepen existence. Participants respond to items using a 7-point Likert scale, ranging from 1 (absolutely untrue) to 7 (absolutely true). Respectively, higher scores denote greater presence and search.

### Social identity

The researchers assessed social identity using a single-item social identification measure (SISI). This approach was consistent with Postmes et al. (2013), who reported that the construct of social identification was sufficiently homogeneous to be adequately operationalized with a single item. The item asked participants to record their response to the statement ‘I strongly identify with the society in which I live’ via a 7-point Likert scale (1 = strongly disagree to 7 = strongly agree). A higher score specifies greater identification.

Measures possessed established psychometric properties: RPBS (Drinkwater et al., 2017; Dagnall et al., 2023a); GCBS-5 (Dagnall et al., 2023b; Kay and Slovic, 2023); SOP2 (Nießen et al., 2022); Brief-COPE (Carver, 1997); MLQ (Steger et al., 2006); and SISI (Postmes et al., 2013).

### Procedure

A web link directed prospective participants to the study brief, which outlined the study (i.e., requirements, procedure, and ethical details). As participants completed the study measures on three occasions instructions asked them to provide an identification number. The researchers deleted this following data collation. Only participants who provided consent progressed to study measures. To ensure standardization across completion waves, instructions remained constant.

The researchers used procedural remedies to reduce the possibility of methodological effects (i.e., common method variance, social desirability, and evaluation apprehension). Specifically, with the exception of the demographic section, which participants always completed first, measure order varied. The researchers hosted the study in Qualtrics, a cloud-based survey platform, and used the inbuilt randomizer function to counter potential order effects. Additionally, the study brief and section headings created psychological separation between constructions by emphasizing measure distinctiveness. Finally, instructions specified that there were no correct answers; participants should read items carefully, respond at their own pace, and complete all sections.

### Ethics statement

The Health and Education Research Ethics and Governance Committee at Manchester Metropolitan University granted ethical approval for this project (ID, 47784).

## Results

### Analysis plan

Prior to testing a path model, data screening and assessment of correlations occurred. The model assessed relationships between Paranormal Belief (PB), Conspiracy (exogenous variables), Optimism, Pessimism, Coping (Active and Avoidant) (mediators), Meaning in Life (MLSearch and MLPresence), and Social Identity (endogenous variables) across multiple time points.

To evaluate standard model fit, the researchers consulted Confirmatory Fit Index (CFI), Standardized Root-Mean-Square Residual (SRMR), and Root-Mean-Squared Error of Approximation (RMSEA). CFI > 0.95, SRMR <0.05, and RMSEA <0.05 designate good data-fit (Hu and Bentler, 1999). Bootstrapping of indirect/mediating relationships occurred to create 95% bias-corrected confidence intervals (1,000 resamples) (Preacher and Hayes, 2008). Model comparison among nested solutions used the Satorra-Bentler chi-square test (Asparouhov and Muthén, 2010).

### Data screening

Assessment of normality revealed no issues; specifically, skewness values fell between −1.0 and +1.0 (Brown, 2015). Intervariable correlations appear in Table 1.

**Table 1:** Descriptive statistics and intercorrelations among study variables.

Variable	*Mean*	*SD*	1	2	3	4	5	6	7	8	9
1. T1 Paranormal Belief	86.97	35.13		0.60**	0.13**	0.10**	0.10**	0.40**	0.13**	0.38**	0.13**
2. T1 Conspiracy	14.75	4.95			0.05	0.14**	0.05	0.31**	0.03	0.27**	−0.02
3. T2 Optimism	4.24	1.53				−0.61**	0.39**	0.01	0.56**	0.03	0.48**
4. T2 Pessimism	3.94	1.60					−0.18**	0.38**	−0.45**	0.19**	−0.28**
5. T2 Active Coping	31.78	7.99						0.09*	0.31**	0.06	0.22**
6. T2 Avoidant Coping	22.11	7.88							−0.09*	0.39**	0.09*
7. T3 MLPresence	21.43	6.86								−0.06	0.49**
8. T3 MLSearch	20.50	7.35									0.05
9. T3 Social Identity	4.22	1.50									

Raw means; T1 = Time 1, T2 = Time 2, T3 = Time 3; * indicates *p* < 0.05, ** indicates *p* < 0.001.

### Path analysis

Path analysis tested predictive relationships between variables. The initial model (Model a) was saturated (i.e., included all construct relationships) and evidenced perfect fit. Accordingly, subsequent model fit evaluation used trimmed versions. Model b with non-significant predictive paths between exogenous and mediator variables constrained to zero (i.e., Conspiracy with Optimism and Active Coping; PB with Pessimism) and Model c, with non-significant paths between exogenous, mediating, and endogenous variables constrained to zero (i.e., Conspiracy and MLPresence; Conspiracy, Optimism, and Active Coping with MLSearch; Pessimism and Active Coping with Social Identity).

Model comparison revealed a non-significant worsening of fit for Model b (vs. Model a), S–B *χ*^2^ = 2.84 (*df* = 3, *p* = 0.416). Fit of Model b was good, χ^2^ (3, *N* = 1,158) = 2.84, *p* = 0.416, CFI = 1.0, SRMR = 0.01, RMSEA = 0.01 (95% CI of 0.01 to 0.04). Model c found non-significant worsening in fit vs. Model b, S–B *χ*^2^ = 6.16 (*df* = 6, *p* = 0.405). Model c provided the superior, parsimonious solution. Model c fit was good, χ^2^ (9, *N* = 1,158) = 9.02, *p* = 0.434, CFI = 1.0, SRMR = 0.01, RMSEA = 0.01 (95% CI of 0.01 to 0.03).

**Figure 1:**
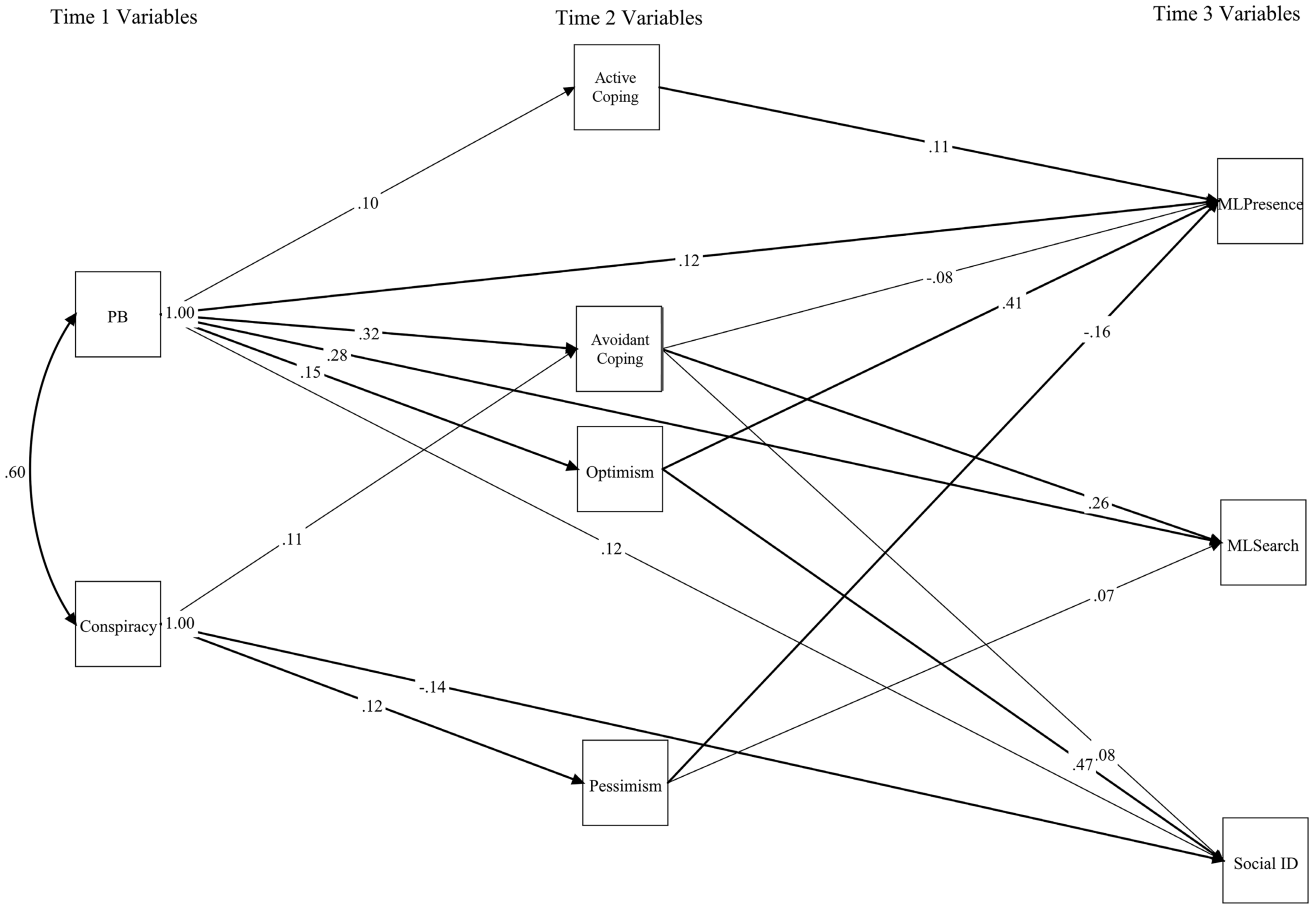
Multiple time point mediation model depicting putative relationships between PB, conspiracy, Optimism, Pessimism, coping, MLPresence, MLSearch, and Social Identity. Standardized regression weights between variables shown. Error not indicated but specified for all variables. Bold arrows depict significant loadings/relationships at *p* < 0.001; faded arrows depict significant loadings/relationships at *p* < 0.05. Analysis used Bootstrapping significance estimates (1,000 resamples). PB, Paranormal Belief; Social ID, Social Identity.

Assessment of relationships signified that PB significantly positively predicted MLPresence, MLSearch, and Social Identity (Figure 1). Conspiracy significantly (negatively) predicted Social Identity only. The model accounted for 36% of MLPresence, 22% of MLSearch, and 25% of Social Identity variance.

Reporting of mediation excluded Conspiracy and Meaning in Life because analysis constrained direct paths to zero. Active Coping and Optimism positively mediated the PB-MLPresence relationship, and Avoidant Coping was a negative mediator (Table 2). This specified that, in the context of PB, Optimism alongside the use of Active Coping was affiliated with greater MLPresence (i.e., strengthened the relationship), whereas Avoidant Coping predicted lower levels of MLPresence. Avoidant Coping positively mediated the relationship between PB and MLSearch, PB and Social Identity (Optimism was an additional positive mediator), and Conspiracy and Social Identity. In the context of PB, use of Avoidant Coping predicted greater MLSearch and Social Identity (i.e., they enhanced the relationship). Optimism additionally enhanced the positive relationship between PB and Social Identity. Moreover, Avoidant Coping was affiliated with higher levels of Social Identity in relation to Conspiracy.

**Table 2:** Indirect effects of paranormal belief and conspiracy on study outcomes through optimism and coping.

Indirect path	MLPresence	MLSearch	Social identity
	*β* (95%CI)	*β* (95%CI)	*β* (95%CI)
Paranormal Belief > Optimism	0.06** (0.04,0.08)	-	0.07** (0.05,0.09)
Paranormal Belief > Active Coping	0.01* (0.01,0.02)	-	-
Paranormal Belief > Avoidant Coping	−0.03** (−0.04,-0.01)	0.08** (0.06,0.11)	0.03* (0.01,0.05)
Conspiracy > Avoidant Coping	-	-	0.01* (0.01,0.02)

* indicates *p* < 0.05, ** indicates *p* < 0.001 using Bootstrapping significance estimates (1,000 resamples).

## Discussion

Examination of zero-order correlations revealed theoretically important relationships between paranormal belief, conspiracy endorsement, and wellbeing measures. As predicted, Paranormal Belief (PB) and Conspiracy strongly positively correlated (see Gignac and Szodorai, 2016). This association concurred with preceding investigations (Drinkwater et al., 2012; Brotherton and French, 2014; Dagnall et al., 2017). The fact that studies, despite using an array of measurement instruments, consistently report an association between the constructs demonstrates the robustness of this affiliation. Particularly, the correspondence shows that PB and Conspiracy share overlapping psychological features. In the present study, these were relationships with non-adaptive psychological factors (i.e., Pessimism, Avoidant Coping, and Search for Meaning in Life; MLSearch). Collectively, these variables reflect an insecure worldview, characterized by perceived lack of volition/control and purpose (Dagnall et al., 2015).

The positive association between PB and Conspiracy, concomitant with the observed attendant correlations with wellbeing measures, provide some support for theorists who locate paranormal belief and conspiracy within a common classification alongside other scientifically questionable/empirically dubious beliefs such as pseudoscience (e.g., Mill et al., 1994; Lobato et al., 2014; van Prooijen et al., 2022). However, it is important that construct overlap is not confused with sameness to the neglect of crucial differences. Hence, though parsimonious and useful to the extent that it denotes the presence of mutual features (e.g., faulty worldview, Jastrzębski and Chuderski, 2022), this generic classification is problematic if applied without qualification.

Even in this study where the correspondence between PB and Conspiracy was at the upper end of the observed range, the constructs shared only 36% variance. Moreover, the finding that only PB was positively related to affirming psychological factors (Optimism; Active Coping; Presence of Meaning in Life, MLPresence; and Social Identity) illustrates the need to assess unorthodox conviction types independently. In the case of PB, credence affords psychological benefits that potentially offset or counterbalance non-adaptive features. This though was not the case with Conspiracy.

Since the two dimensions of the Meaning in Life Questionnaire are differentially linked to psychological health, subscales scores illustrate this point. Search allies with reduced wellbeing and negative affect (e.g., sadness and rumination) (Dakin et al., 2021), whereas presence relates to life satisfaction and is inversely associated with negative factors (e.g., depression) (Steger et al., 2006). Search though can prove constructive when it enhances or culminates in perceived sense of purpose (i.e., affords reassurance by resolving uncertainty and/or providing solutions) (Newman et al., 2018). The outcomes of this study suggest that this is true of PB but not Conspiracy.

Recent work on synchronicity supports the supposition that search is attendant with positive wellbeing through presence (Russo-Netzer and Icekson, 2023). Synchronicity refers to unusual and meaningful coincidences that represent something more than mere chance. Particularly, these occurrences link the internal and external worlds of the individual (Jung, 1969). The synchronicity model, drawing on the meaning-as-information approach (Heintzelman and King, 2014), specifies that search increases openness to synchronicity events anomalous beliefs/phenomena (i.e., anomalous beliefs/phenomena). This in turn aids comprehension and provides meaning and optimism, which contributes to greater life satisfaction. Within the model, increased optimism/positive future perspective (i.e., positive emotion) is concomitant with heightened sense of purpose (Steger et al., 2011). Findings from the present study concur with preceding research and designate that search for meaning in life positively contributes to life satisfaction when it provides personal understanding and sense of purpose (Russo-Netzer, 2019).

Extrapolating this notion to coping and outlook a similar pattern of results emerged. Explicitly, PB was related to both psychologically maladaptive (Avoidant Coping and Pessimism) and adaptive (Active Coping, Optimism, and Social Identity) features. Contrastingly, Conspiracy only positively correlated with psychologically maladaptive (Avoidant Coping and Pessimism) features. Overall, divergence on positive attributes (i.e., Presence, Active Coping, Optimism, and Social Identity) tentatively indicated that PB is concurrently attendant with passive and active psychological functions. From this perspective, PB is best conceptualized as both a reaction and a response to life uncertainties. Explicitly, active elements afford personal reassurance that counters perceived uncertainty and lack of control. This active, dynamic component is absent within conspiracy. Hence, conspiracy beliefs consolidate/confirm rather than negate or protect against existential concerns.

The path model also revealed differences between PB and Conspiracy. Explicitly, Active Coping and Optimism positively mediated the PB-MLPresence relationship. Avoidant Coping was a negative mediator. Additionally, Avoidant Coping positively mediated the PB and MLSearch, PB and Social Identity (Optimism was an additional positive mediator), and Conspiracy and Social Identity relationships. This indicated that Active Coping and Optimism enhanced the PB and MLPresence relationship, whereas Avoidant Coping contributed to lower MLPresence. Correspondingly, Avoidant Coping increased the strength of the relationship between PB, MLSearch and Social Identity. Avoidant Coping had a similar effect on the Conspiracy-Social Identity relationship. These results indicate that PB and Conspiracy relate differently to wellbeing related outcomes, and additionally reinforce the observation of Dagnall et al. (2024b) that PB influences factors associated with positive wellbeing (e.g., meaning in life) via its link with coping (avoidant and active).

The connection with avoidant coping aligns with the notion that paranormal belief equivocates management of difficult feelings and situations (Dagnall et al., 2022c). In turn, avoidant coping is associated with lower wellbeing (e.g., distress) and experience of distress/lower wellbeing, which as a consequence of existential angst/uncertainty, motivates search for meaning in life as Cohen and Cairns (2012). In this context, the association between avoidant coping and scientifically unverified beliefs stems from disengagement with critical thinking and the tendency to seek purpose and reassurance in unverified narratives (Leman and Cinnirella, 2013).

This may explain why avoidant coping is concurrently linked to search for meaning in life. Collectively, findings suggest that avoidance reflects underlying uncertainty, thereby increasing the appeal of supernatural or conspiratorial explanations (Routledge et al., 2017). Furthermore, as individuals retreat into ideologically homogeneous groups that validate their worldview and mitigate anxiety, avoidant coping reinforces the role of social identity in shaping these beliefs (van Prooijen et al., 2022).

Conversely, Active Coping and Optimism serving to enhance the PB-MLPresence relationship indicates a more adaptive path, facilitated by possession of a positive outlook and a motivation to resolve difficulties (Steger et al., 2008). The association of PB with both avoidant and active coping as precursors of differential wellbeing outcomes warrants further research, however, to elucidate underpinning mechanisms.

## Limitations

The present paper considered only a narrow range of subjective wellbeing outcomes. Noting this, subsequent studies should conduct further comparisons of the adaptive functions of paranormal belief (vs. conspiracy) using a broader range of psychological health-related indices. This will establish the extent to which the two constructs diverge and influence psychological health/adjustment. Moreover, the present study used only hedonic indices (i.e., emotional balance). Acknowledging this constraint, future work should include eudaemonic (i.e., emotional health) measures. Though widely used as an outcome measure, hedonic wellbeing provide only limited insights into wellbeing because of the concept’s focus on the pursuit of satisfaction/pleasure and the absence of negative emotion. While these are important attributes, beliefs that evoke positive emotions or possess affirming connotations may facilitate high-order psychological processes (e.g., self-fulfillment & realization). These wellbeing elements are fundamental to the eudaemonic approach, which proposes that positive emotions enable optimal functioning and personal enrichment (i.e., growth, authenticity, autonomy, relationships, & environmental mastery).

A further shortcoming of this study was the use of a general measure of paranormal belief. While dimensions of supernatural credence correlate highly, evidence suggests that factors vary in terms of adaptivity and emotionality (Lasikiewicz, 2016). Cognizant of this, proceeding scholarly work should examine differences between belief types. For instance, religion provides existential meaning, purpose, and social support (i.e., structured worldview), whereas paranormal belief generally lacks a cohesive meaning making framework (Aarnio and Lindeman, 2005). Thus, while both constructs afford existential reassurance, research reports that religious belief, due to its communal and moral dimensions, is more strongly positively associated with wellbeing and life satisfaction (Schnell, 2012).

Hence, consideration of belief types will provide a more nuanced understanding of the interaction between supernatural credence, emotion, and wellbeing. Explicitly, it will identify and facilitate the integration of affirming beliefs into wellbeing models such as broaden-&-build theory (Fredrickson, 2004) and PERMA (positive emotion, engagement, relationships, meaning, & accomplishment; see Seligman, 2018). These models, via positive emotions, suggest mechanisms by which paranormal beliefs can enhance wellbeing. Moreover, they can inform the advancement of testable paranormal belief-based models similar to the one proposed for synchronicity (Russo-Netzer and Icekson, 2023).

Regarding conspiracy theory, conclusions require cautious interpretation because the study assessed endorsement using a unidimensional measure. Though, given the absence of life affirming and positive indicators, it is unlikely that conspiracy theory enhances subjective wellbeing, consideration of variations across factors may prove informative. Explicitly, identify the facets of advocacy that are most strongly attendant with lower levels of adaptive functioning. Accordingly, follow-up investigations could use GCBS dimensions (i.e., Government Malfeasance, allegations of routine criminal conspiracy within governments; Extraterrestrial Cover-up, public deception concerning the existence of aliens; Malevolent Global Conspiracies, allegations that small, secret groups exert total control over world events; Personal Wellbeing, concerns over personal health and liberty such as the spread of diseases and the use of mind-control technology; and Control of Information, unethical manipulation and suppression of information by organizations).

## Data Availability

Raw data supporting the conclusions of this article will be made available by the authors, without undue reservation.
